# Prolonged Pediatric Extracorporeal Membrane Oxygenation Support with Cardiopulmonary Failure in Juvenile Myelomonocytic Leukemia

**DOI:** 10.1155/2020/5696380

**Published:** 2020-01-13

**Authors:** Maris Bartkevics, Bert Hennig, Tayfun Gungor, Roland Ammann, Alexander Kadner

**Affiliations:** ^1^Center for Congenital Heart Disease, Department of Cardiovascular Surgery, Inselspital University Hospital, Bern University, Bern, Switzerland; ^2^Department of Pediatrics, University Children's Hospital, Bern University, Bern, Switzerland; ^3^Department of Stem Cell Transplantation, University Children's Hospital Zurich, University Zurich, Zürich, Switzerland

## Abstract

We report a case of a child survival after extracorporeal membrane oxygenation (ECMO) support of 25 days for cardiopulmonary failure and septic shock in the context of juvenile myelomonocytic leukemia (JMML). ECMO support is still a matter of debate for the management of septic patients with malignancy. However, these patients are at increased risk for early death secondary to pulmonary complications due to leukostasis, direct pulmonary infiltration with WBC, and systemic inflammatory response syndrome following malignant cell lysis. Despite the high risk of complications, ECMO support must be discussed as part of management, providing better outcome in this group of patients.

## 1. Introduction

Extracorporeal membrane oxygenation (ECMO) support is still a matter of debate for the management of septic patients with malignancy. However, these patients are at increased risk for early death secondary to pulmonary complications due to leukostasis, direct pulmonary infiltration with WBC, and systemic inflammatory response syndrome following malignant cell lysis [1].

We report a case of successful management of a child with juvenile myelomonocytic leukemia (JMML), who required prolonged ECMO support secondary to cardiopulmonary failure following initiation of chemotherapy.

## 2. Patients' Medical Report

A 4-year-old boy presented with an intermittent fever for two weeks, cold symptoms, and loss of appetite. On physical examination, the child was found moderately dehydrated, tachycardic, and tachypneic and demonstrated a hepatosplenomegaly. His peripheral blood tests showed 10% myeloid blasts with WBC (16.2 × 10^9^/L), severe anemia (Hb 2.8 g/dL), and thrombocytopenia (platelets 6 × 10^9^/L), as well as a monocyte count of 4.61 × 10^9^/l. A bone marrow aspiration was performed and revealed 10% of myeloid blast cells. The patient was started on an induction regimen with cytarabine and rasburicase. Less than 24 hours after initiation of therapy, he developed fever and progressive respiratory failure. A chest X-ray showed a bilateral pulmonary infiltration. In the context of suspected sepsis, the broad-spectrum antibiotic treatment was started. Echocardiography showed signs of severe pulmonary hypertension (PHT). Despite inotropic support, mechanical ventilation, and early antibiotics, the patient deteriorated and was referred to our tertiary pediatric intensive care unit.

**Figure 1:**
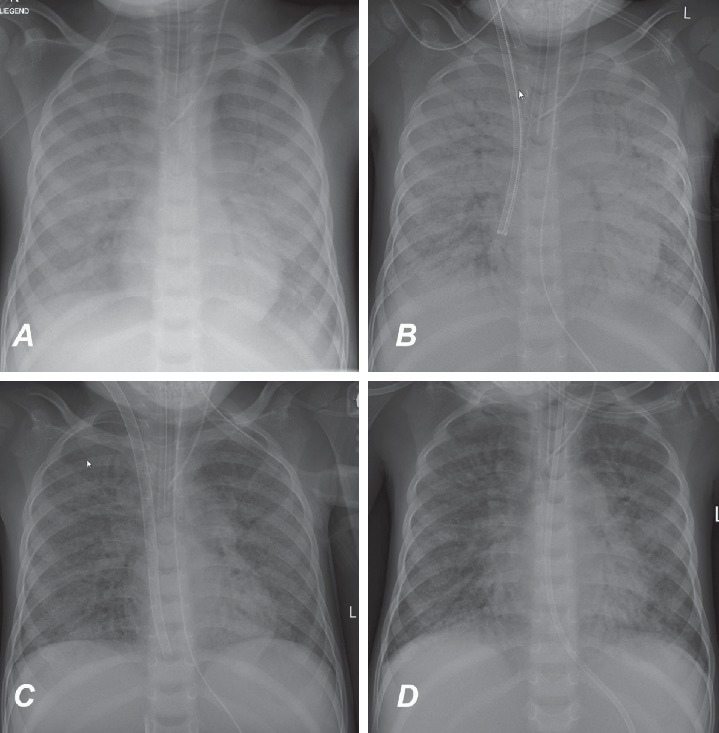
(a) Chest X-ray on admission. (b) Chest X-ray after ECMO cannulation via neck vessels. (c) Chest X-ray after conversion to veno-venous ECMO by using single venous neck cannula. (d) Chest X-ray after ECMO weaning.

**Figure 2:**
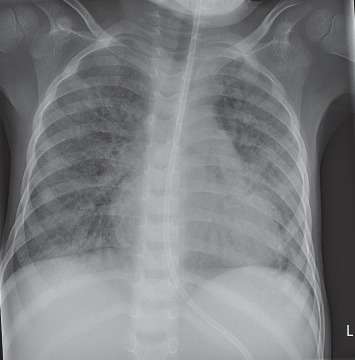
Chest X-ray after extubation.

At arrival, his general condition was critical with signs of multiorgan failure and severe lactic acidosis. The neurological status appeared to be normal. His acute respiratory distress syndrome necessitated high-pressure ventilation (Figure 1(a)). Despite optimized conventional therapy, he rapidly developed cardiogenic shock. A veno-arterial (VA) ECMO was initiated via neck vessel cannulation, and immediate improvement in oxygenation was achieved (Figure 1(b)). ECMO flow was stable between 1.2 – 1.7 L/min during the whole support period. ACT was kept at 180 seconds. No major bleeding complications occurred. Mechanical ventilation was continued, and PHT therapy was initiated with sodium nitroprusside and nitrous oxide. First improvements in ventilation were observed on day 12 on ECMO support. On day 18, VA ECMO could be converted to a veno-venous (VV) ECMO by using a dual lumen Avalon ELITE cannula (MAQUET Cardiopulmonary AG, Germany) (Figure 1(c)). After seven days of VV ECMO support, the patient was successfully weaned on day 25 (Figure 1(d)). The neurologic assessment of the patient was limited during ECMO support due to pharmacological sedation. Following further hemodynamic improvement and weaning of sedation, the patient demonstrated a quadriplegia, global aphasia, and conjugate eye deviation. Subsequent MRI diagnostic revealed a central pontine myelinolysis. Certainly, it was caused by dyselectrolaemia with severe hypernatremia. Despite severe neurological impairment, his respiratory condition improved further, and 2 days later (day 30), he was extubated (Figure 2). Following extubation, a bilateral vocal cord paralysis of probably central origin was noticed.

Given the fast improvement of the patient, the initially paused chemotherapy with cytarabine was restarted early, thus allowing a cytogenetic remission on day 21 of ECMO support.

On day 34 of hospitalization, the patient was referred for evaluation for allogeneic hematopoietic stem cell transplantation to the transplantation center. A genetic testing revealed a KRAS and SETBP1 mutation. Given the severe neurological impairment, the decision was taken to proceed with bridging chemotherapy until improvement of the neurological status could be observed.

Five weeks later, the patient's clinical condition was normal with a remarkably improved neurological status and normalized vocal chord function. The patient could walk, even drive a 3-wheel bike, while his cognitive function was still impaired with a stammer requiring further speech and language therapy.

Six months later, he underwent allogeneic hematopoietic stem cell transplantation with a 10/10 HLA-matched unrelated donor donating bone marrow. The conditioning comprised fludarabine, targeted busulfan, and melphalan including rabbit ATG. He engrafted at day +26 and +23 with neutrophils and platelets of donor origin. The child was discharged home with minor cognitive impairment.

## 3. Discussion

To our knowledge, this is the first report of survival after prolonged ECMO support (25 days) in a child with JMML and cardio-respiratory failure secondary to tumor lysis syndrome. Meister et al. published a case report of an infant with JMML, who was on ECMO support for 5 days and died because of pulmonary failure [2]. JMML is a rare, aggressive, clonal hematopoietic disorder of childhood characterized by overproduction of monocytic and granulocytic cells with organ infiltration, such as spleen, liver, and lungs [3]. A life-threatening complication is the occurrence of a TLS arising most commonly after initiation of chemotherapy. Mortality is reported between 21–66% [4]. Generally, the main principles of management include early recognition of metabolic and renal complications and the prompt administration of inotropic and respiratory support. However, there are no data and recommendations on the application of ECMO therapy in those cases. Considering that in JMML hemorrhagic and thrombotic complications are frequent and survival for refractory shock in pediatric patients with or without sepsis is limited, the initiation of ECMO therapy is debatable and might be withheld from those patients. The reported case demonstrates that successful management of cardiorespiratory failure in the setting of TLS in JMML can be achieved. Early administration of ECMO should be considered and allows for immediate stabilization of cardiorespiratory function in this context. Despite the high risk of hemorrhagic and thromboembolic complications in patients with JMML, prolonged ECMO support should be attempted.

## Abbreviations

ECMO: Extracorporeal membrane oxygenation
JMML: Juvenile myelomonocytic leukemia
PHT: Pulmonary hypertension
VA: Veno-arterial
VV: Veno-venous
WBC: White blood cells.
